# Development and evaluation of a high-fidelity lactation simulation model for health professional breastfeeding education

**DOI:** 10.1186/s13006-020-0254-5

**Published:** 2020-02-17

**Authors:** Anna Sadovnikova, Samantha A. Chuisano, Kaoer Ma, Aria Grabowski, Kate P. Stanley, Katrina B. Mitchell, Anne Eglash, Jeffrey S. Plott, Ruth E. Zielinski, Olivia S. Anderson

**Affiliations:** 1 LiquidGoldConcept, Inc., 124 Pearl St Suite 404, Ypsilanti, MI 48197 USA; 2 Graduate Group in Nutritional Biology, Physician Scientist Training Program, University of California, Davis, Davis, CA USA; 3 Department of Nutritional Sciences, University of Michigan School of Public Health, Ann Arbor, MI USA; 4 Division of Neonatal-Perinatal Medicine, Department of Pediatrics, University of Michigan Medical School, Michigan Medicine, Ann Arbor, MI USA; 5 Department of Surgical Oncology, Ridley Tree Cancer Center at Sansum Clinic, Santa Barbara, CA USA; 6 University of Wisconsin School of Medicine and Public Health, Madison, WI USA; 7 Department of Mechanical Engineering, University of Michigan, Ann Arbor, MI USA; 8 Department of Health Behavior and Biological Sciences, School of Nursing, University of Michigan, Ann Arbor, MI USA

**Keywords:** Breastfeeding education, Lactation simulation model, Breast model, Breastfeeding simulator, Medical education, Nursing education, Midwifery education, Graduate medical education, High-fidelity, Clinical lactation

## Abstract

**Background:**

A key reason for premature cessation of breastfeeding is inadequate support from healthcare providers. Most physicians and nurses do not feel confident in their ability to support families with breastfeeding initiation or maintenance. Increasing health professional confidence in clinical lactation skills is key to improving maternal and child health outcomes. High-fidelity (realistic) simulators encourage learner engagement, resulting in increased clinical skills competency, confidence, and transfer to patient care. Lactation educators teach with low-fidelity cloth and single breast models. There are no high-fidelity breast simulators for health professional education in clinical lactation.

**Development and evaluation of a high-fidelity lactation simulation model:**

In this commentary we describe the development of a high-fidelity Lactation Simulation Model (LSM) and how physician residents, nurse-midwifery students, and clinical lactation experts provided feedback on LSM prototypes.

**Limitations:**

The user-testing described in this commentary does not represent comprehensive validation of the LSM due to small sample sizes and the significant conflict of interest.

**Conclusion:**

For breastfeeding rates to improve, mothers need support from their nurses, midwives, pediatricians, obstetricians and gynecologists, and all healthcare staff who interact with pregnant and lactating women. Clinical education with high-fidelity breastfeeding simulators could be the ideal learning modality for trainees and hospital staff to build confidence in clinical lactation skills. The ability of a high-fidelity breastfeeding simulator to increase a learner’s lactation knowledge and psychomotor skills acquisition, retention, and transfer to patient care still needs to be tested.

## Background

Low maternal breastfeeding self-efficacy and inadequate lactation support from healthcare providers are key reasons for premature breastfeeding cessation [1–3]. Insufficient clinical education in lactation support is a longstanding problem across healthcare specialties, professions, and levels of training [4–11]. Most physicians and nurses do not feel confident in their ability to support families with breastfeeding initiation or maintenance [6–10]. Nursing and medical students are rarely exposed to breastfeeding mothers during clinical rotations [6–13]. If students do interact with breastfeeding patients, they are usually shadowing a lactation specialist and do not have the time or confidence to practice breastfeeding skills [6–13].

Educators use simulation for learners to engage in maternal-child patient care situations they would otherwise rarely encounter during training to promote technical and non-technical skills development, decrease learner anxiety, and improve patient safety and health outcomes [13–16]. The World Health Organization strongly recommends the use of “high-fidelity” (realistic) simulation for health professional education because it leads to greater acquisition, retention, and transfer of technical and non-technical skills [17]. Low-fidelity commercially-available or handmade cloth breast models are frequently used in breastfeeding education, but the approach is not standardized and learning and patient outcomes are rarely assessed [5, 11, 12]. We propose that high-fidelity simulation is the ideal learning modality for breastfeeding education for three reasons:

1. Lactation support requires deliberate practice and confidence in examining, touching, and moving breast tissue. Since breasts are an intimate body part, a safe learning environment could facilitate the development of core breastfeeding skills. Hand expression of breastmilk, breast examination, breast massage, and newborn positioning and attachment at the breast all require confidence in using ones’ hands to touch and move breast tissue [5, 16, 18, 19].
2. The postpartum period is a vulnerable time for new mothers [20]. Real patients experiencing breastfeeding challenges could feel overwhelmed when groups of trainees are brought into the patient room for clinical learning. A hybrid simulation approach would allow for learners to deliberately practice empathetic and culturally-competent counseling in a variety of clinical lactation case scenarios [13, 21, 22].
3. Required clinical rotations in nursing, midwifery, and medical school do not always provide students the opportunity to interact with diverse breastfeeding patients. As a result, most healthcare providers do not have experience identifying or managing common breastfeeding complications. A hands-on workshop with high-fidelity breast simulators depicting diverse nipple-areolar complex anatomy, dermatoses, or breast surgical scars would provide medical, midwifery, and nursing school graduates with a well-rounded education in breast health and lactation [6–9, 11, 13, 14].

Industry stakeholders in healthcare simulation are passionate about patient safety and healthcare quality improvement [23]. While research and development efforts consume a substantial portion of a company’s revenue, study results are rarely published [23]. Only 6.5% of commercially-available simulators have been assessed for face or content validity, meaning that very few studies have been published describing the evaluation of a product’s appropriateness or realism [24]. While the development and evaluation of high-fidelity breast simulators for surgical training has been described, there are no published studies describing user-testing of commercially-available breastfeeding simulators [25, 26].

Here we first describe how the user requirements for a breastfeeding simulator’s form and function were established in 2015. We used the user requirements to develop a Lactation Simulation Model (LSM) prototype suitable for testing in 2017. Between 2017 and 2018 we developed the market-ready Essential and Advanced LSMs. Feedback on the LSMs’ realism and functionality was obtained from three user groups: 1) resident physicians in obstetrics and gynecology and family medicine at the University of Michigan, 2) nurse-midwifery students at the University of Michigan, and 3) breastfeeding medicine specialists at a symposium led by the Institute for the Advancement of Breastfeeding and Lactation Education. All users performed a breast assessment on a LSM prototype, drew features they identified on a breast line drawing, and rated the realism of experience and the LSM’s look, feel, and functionality by answering closed-ended (defined, 7-point Likert scale) and open-ended questions in a LSM Questionnaire. From the three user tests, the manufacturer obtained the following information: 1) do the LSM’s breast tissue and lactation-related conditions look and feel realistic, 2) is the experience of performing hand expression on engorged and non-engorged breasts of the LSM realistic, 3) is the experience of using a breast pump with the LSM realistic, and 4) can users identify normal and abnormal features on the LSM?

### Establishing the user requirements for a breastfeeding simulator’s form and function

**Fig. 1:**
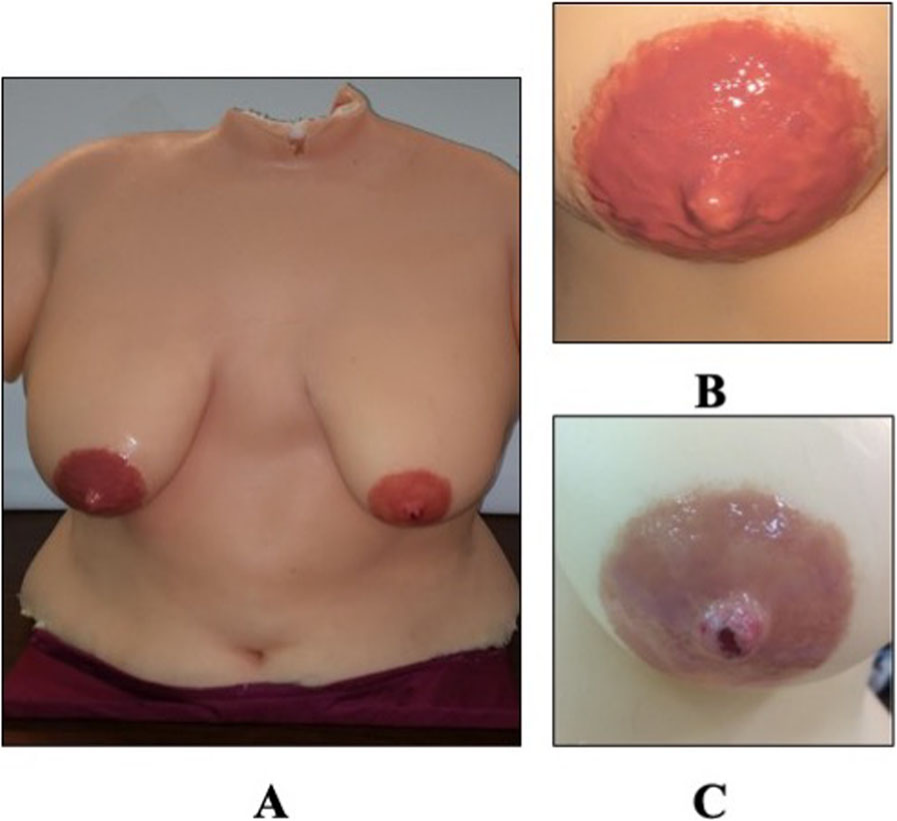
Description of features on the LSM proof of concept (2015). **a** 2015 LSM Proof-of Concept **b**. Round nipple on right breast **c**. Nipple with damage on the left breast

In 2015 the manufacturer developed a LSM proof-of-concept (Fig. 1) under the guidance of the company’s breastfeeding medicine advisor, a board-certified pediatrician, lactation consultant, and a fellow of the Academy of Breastfeeding Medicine with over two decades of experience working with breastfeeding dyads.

The manufacturer created a survey (Additional file 1**)** to define the common clinical lactation skills an educator would like to teach with a LSM. The survey contained questions about respondents’ personal and professional breastfeeding experiences and close-ended (defined, 7-point Likert scale) and open-ended questions about desired LSM form and function. Items within the survey were based on clinical lactation skills identified in Baby-Friendly Hospital “Step 2″ educational guidelines [12].

Five physicians (*N* = 5) at the 2015 Academy of Breastfeeding Medicine conference completed the user requirement survey. All five respondents had personal and/or professional breastfeeding experience and three respondents had provided breastfeeding education to health professional students. The respondents agreed that a breastfeeding simulator could be a valuable (6.6/7) and relevant (6.0/7) training tool. Respondents preferred a wearable LSM shaped like a torso instead of a single breast, realistic look and feel of breast tissue, and a diversity of nipple shapes and sizes. The most important capabilities selected by all respondents were the ability to demonstrate hand expression, massage for engorgement or plugged ducts, use a breast pump, and identify sore, cracked, or bleeding nipples.

The manufacturer set out to create a LSM prototype suitable for testing that satisfied the user requirements defined in 2015. Novel internal components for lactation, engorgement, and plugged duct simulation were designed, a blend of silicone materials was created to better represent the look and feel of breast tissue, and a new mold from a postpartum breastfeeding woman was developed. Nipple damage was illustrated on the left nipple so that an educator could teach about a deep and shallow latch. After 2 years of prototyping and internal testing by the manufacturer’s CEO and breastfeeding medicine advisor, the first LSM prototype was ready for user feedback in June 2017.

### User-testing with obstetric and gynecology and family medicine physician residents at the University of Michigan

**Fig. 2:**
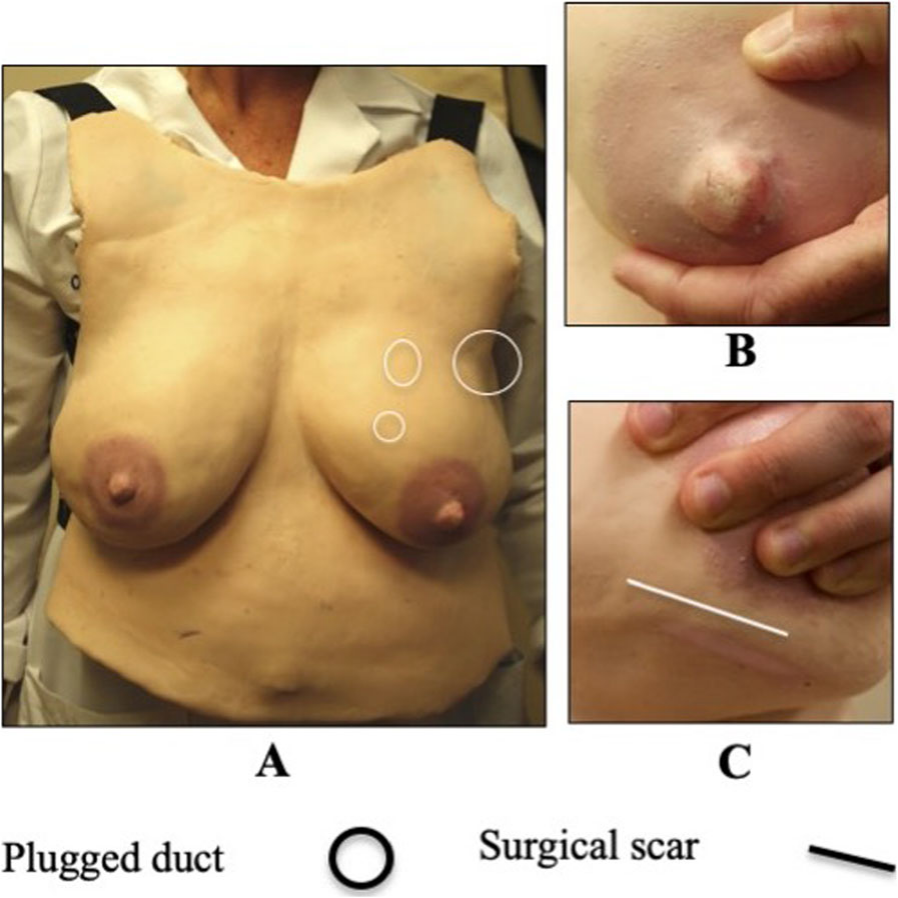
Description of features on the LSM prototype used with obstetrics and gynecology and family medicine residents in 2017. **a** 2017 LSM Prototype. **b** Pinched left nipple with damage. **c** surgical scar on the left breast

The LSM prototype (Fig. 2**)** was incorporated into a prenatal breastfeeding assessment workshop for first year obstetric and gynecology and family medicine residents (*N* = 17) at the University of Michigan in June 2017.

During a 50-min session the residents learned basic lactation physiology and anatomy, used the LSM prototype to practice a breast examination, discussed two case-based clinical scenarios in lactation, and completed the LSM Questionnaire (Additional file 2). This round of user-testing was approved by the University of Michigan Institutional Review Board (HUM00125612).

The majority (88%) of physician residents had never or only sometimes provided breastfeeding education to patients and were not sure of their ability to perform a prenatal breast assessment, provide breastfeeding education, or to identify breast pathologies. Participants agreed the LSM’s nipples and breast tissue (5.5/7) looked and felt realistic. The majority (79%) of physician residents identified the large plugged duct and the scar when performing a breast assessment. Participants agreed (5.9/7) the LSM allowed them to practice comfortable positioning and movement of their hands during a breast examination and helped them learn how to perform a breast assessment (5.7/7).

The main suggestions were to improve the smoothness of the sides of the LSM, make the skin feel less plastic-like and nipples feel less rubbery, improve illustration techniques for the areolae, and add more variation in nipple shapes and sizes. Based on this feedback, the manufacturer improved the manufacturing and illustration techniques and created two new LSM prototypes, an Essential LSM and an Advanced LSM, so that four nipple shapes and sizes and a wide variety of features could be represented.

### User-testing with nurse-midwifery students at the University of Michigan

**Fig. 3:**
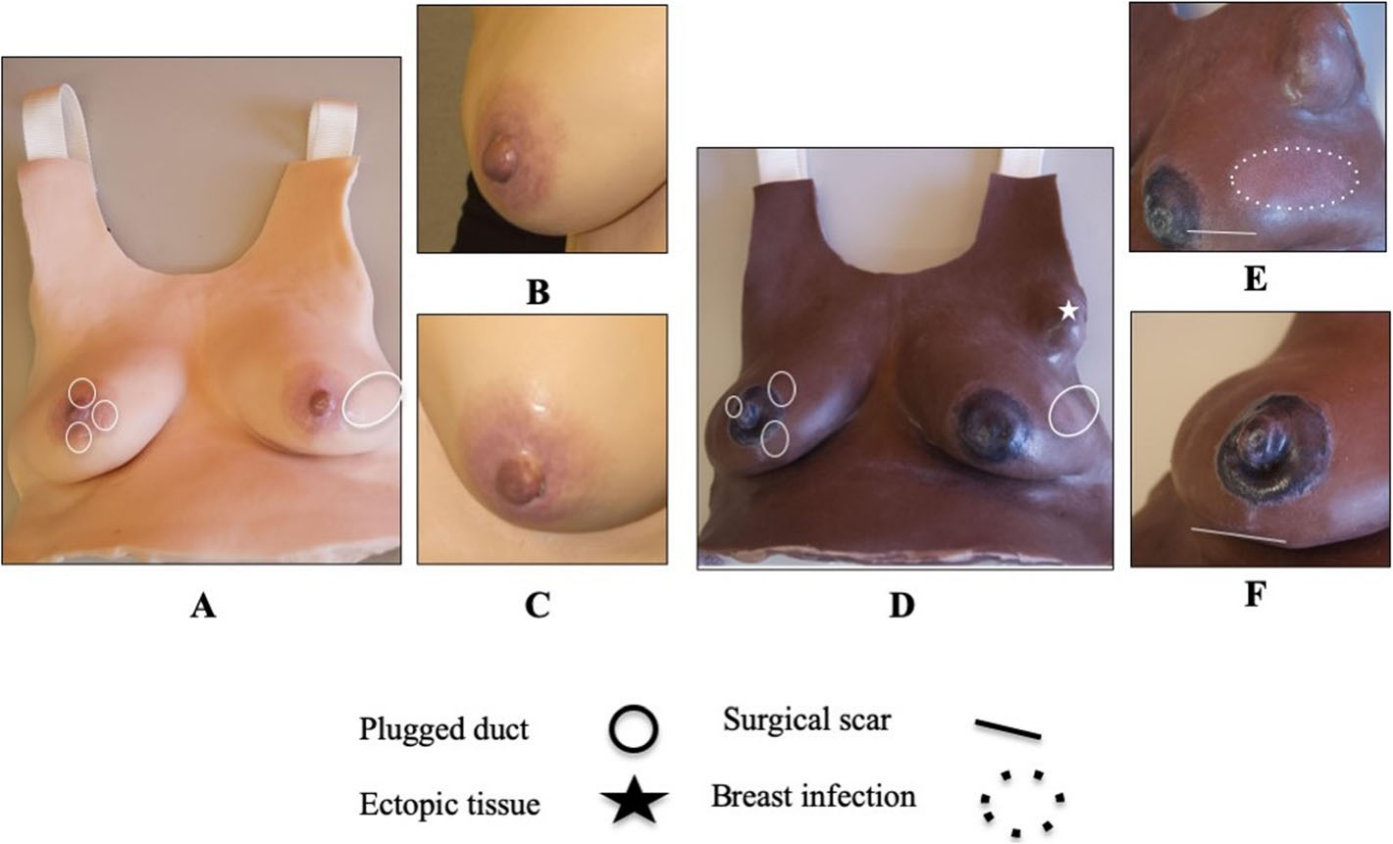
Description of the Essential and Advanced LSM prototypes used with nurse-midwifery students in 2017. **a** Essential LSM in light skin tone. **b** The right breast has a round nipple without damage. **c** The left breast has a pinched nipple with damage. **d** Advanced LSM in dark skin tone. **e** The left breast depicts a flat nipple, augmentation scar, mastitis, and axillary ectopic tissue. **f** The right breast depicts a bulbous nipple, Montgomery glands, and breast reduction scar. Location of features is described in the figure legend

The manufacturer and collaborators at the University of Michigan School of Nursing created two 3-h breastfeeding workshops consisting of two lectures and eight clinical lactation skills cases. Students were asked to complete the LSM questionnaire and worksheets (Additional file 3) to inform the manufacturer about the look, feel, and realism of the new Essential and Advanced LSM prototypes **(**Fig. 3).

The study investigators obtained consent from 12 of the 15 nurse-midwifery students for retrospective analysis of collected data. Repeated measures analysis was possible for nine students. The University of Michigan Institutional Review Board approved the secondary analysis of existing data (HUM00148905).

Most students (7/9) had significant clinical or personal breastfeeding experience. All of the students had performed a breast examination and provided breastfeeding education to patients. Students agreed that both LSMs looked like a breastfeeding mother’s chest both when engorged (6.3/7) and not engorged (6.5/7), but were not sure (4.3/7) if the skin felt realistic. The way that the breast tissue moved in a breast pump (5.7/7) and the way that simulated milk was hand expressed (5.0/7) were deemed realistic. All of the students correctly identified a scar in the left inframammary fold and a periareolar scar on the right breast. They agreed that ectopic tissue looked (5.3/7) and felt (5.2/7) like breast tissue. All students identified different nipple shapes and sizes, plugged ducts, and red discoloration on breast tissue. Throughout the questionnaire and case worksheet, each of the nine students indicated that they were not sure about the realism of the look or feel of some pathologies, likely reflecting differences in prior personal or professional breastfeeding experiences.

The main feedback was to improve the illustration of scars. Students liked that they could practice hand expression and pumping because fluid “actually came out” and appreciated how realistic the LSMs looked and felt. They enjoyed taking turns wearing the product. In response to this feedback, the manufacturer hired medical illustrators to develop four culturally-appropriate skin tones and to ensure that pathologies (e.g. scars) would be represented with higher fidelity.

### User-testing at the Institute for the Advancement of Breastfeeding and Lactation Education clinical case symposium

**Fig. 4:**
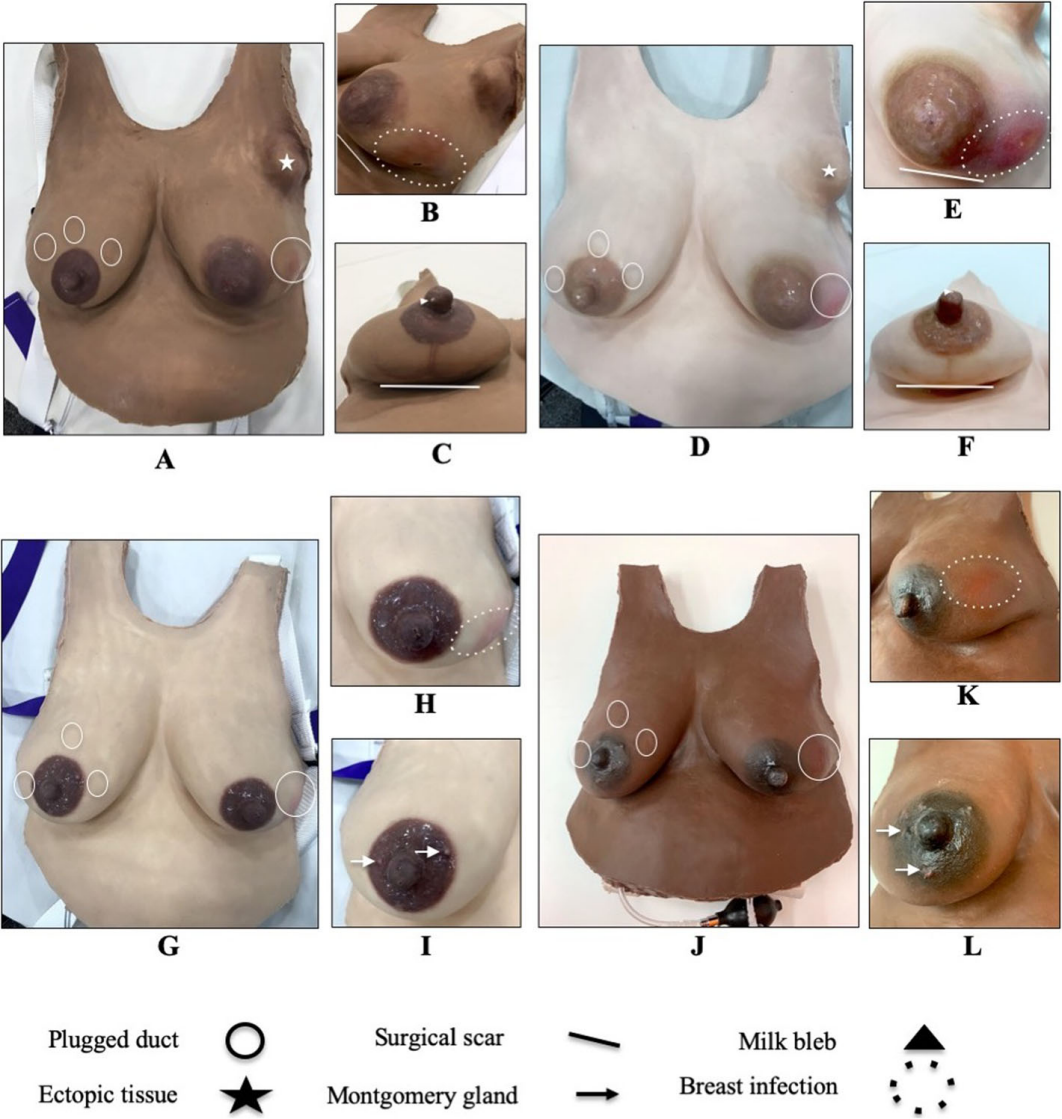
Description of the Essential and Advanced LSM prototypes used with clinical lactation experts in 2018. **a**-**c** Advanced LSM in skin tone **d**-**f**. Advanced LSM in skin tone **g**-**i**. Essential LSM in skin tone **j**-**l**. Essential LSM in skin tone. The features on each LSM are described in the figure legend

The manufacturer provided two new Essential LSMs and two new Advanced LSMs in four skin tones (Fig. 4) for evaluation at a July 2018 symposium led by the Institute for the Advancement of Breastfeeding and Lactation Education. Nine breastfeeding medicine physicians and one non-physician lactation consultant (*N* = 10, “experts”) completed a LSM questionnaire (Additional file 4). The experts had on average 11.7 years of experience with clinical lactation and held or were working towards certifications in lactation. Experts performed a breast examination, rated the realism of the LSM look and feel, provided a diagnosis for each finding, performed hand expression on engorged and non-engorged breasts, and used a breast pump with the LSMs. The University of Michigan Institutional Review Board deemed this study exempt from review (HUM00148728). The study sponsor offered a $10.00 Amazon gift card for questionnaire completion.

Experts agreed that the look and feel of breast tissue (6.1/7) and lactation-related conditions (5.7/7) was realistic. Hand expression (5.4/7) was realistic. Nipple movement in the breast pump flange (5.5/7) and simulated fluid extraction by pump (5.8/7) was realistic, but only when the breast pump suction was strong enough. On the Essential LSM, most experts identified the large plugged duct (70%), nipple damage (80%), and mastitis (60%) and some experts identified at least one of the small plugged ducts (30%) and Montgomery glands (30%). On the Advanced LSM, most experts identified nipple damage (70%), milk bleb (90%), necrosis within the abscess (80%), ectopic breast tissue (100%), periareolar scar (50%), and anchor scar (100%). Experts agreed (6.2/7) that the LSMs could be useful for health professional student, hospital staff, and patient education.

The main suggestions were to soften the breast tissue to make hand expression easier, modify the nipple tissue for better expansion in the breast pump flange, and reduce the simulator’s weight so that it is more comfortable to wear and easier to transport. Since most experts used the highest breast pump settings to see realistic movement of the LSM, we hypothesize that electric, hospital grade pumps would be the best option for educators when teaching breast pump use with the LSMs.

## Conclusions

### Overview

We have described how the Lactation Simulation Models (LSM) were used in educational settings by physician residents and nurse-midwifery students and the feedback that these trainees provided to the manufacturer. Clinical lactation experts agreed that performing basic breastfeeding skills like the breast examination, hand expression, and pumping with the Essential and Advanced LSMs was realistic. For breastfeeding rates to improve in the United States, women need support from their nurses, midwives, pediatricians, obstetricians and gynecologists, and other healthcare providers. Clinical education with high-fidelity breastfeeding simulators is an ideal learning modality for trainees and hospital staff to build confidence in clinical lactation skills. Lactation simulation education has the potential to improve clinical practice and patient outcomes.

### Limitations

The user-testing described in this commentary does not represent comprehensive validation of the LSMs. Sampling was inadequate and it was not possible to perform inferential statistics. The study sponsor was involved in study design and data analysis so there is significant conflict of interest and bias.

### Future research needs

Future unbiased studies are needed to test the LSMs’ ability to increase a learner’s lactation knowledge and psychomotor skills acquisition, retention, and transfer to patient care.

## Supplementary information

**Additional file 1.** User requirement survey (2015). Participant personal and professional breastfeeding background and user requirement survey used to define ideal form and function of a lactation simulation model.

**Additional file 2.** Physician resident survey (2017). Participant personal and professional background LSM validation questionnaire used with obstetrics and gynecology and family medicine residents.

**Additional file 3.** Nurse-midwifery student survey (2017). Participant personal and professional breastfeeding background and LSM validation questionnaire used with nurse-midwifery students.

**Additional file 4.** Clinical lactation expert survey (2018). Participant personal and professional background and LSM validation questionnaire used with clinical lactation experts.

## Data Availability

The datasets used and/or analyzed during the current study are available from the corresponding author on reasonable request.
