# Supplementary material for: Development and evaluation of a high-fidelity lactation simulation model for health professional breastfeeding education
Source: Int Breastfeed J. 2020 Feb 17;15:8. doi: 10.1186/s13006-020-0254-5 (PMC7026968; doi:10.1186/s13006-020-0254-5)
Supplement: Supplementary file 1 — Additional file 1. User requirement survey (2015). Participant personal and professional breastfeeding background and user requirement survey used to define ideal form and function of a lactation simulation model. [file 13006_2020_254_MOESM1_ESM.pdf]

**LGC Breast Massage & Hand Expression Questionnaire 2015**

*Target Participant:* Breastfeeding Expert *Reimbursement:* One raffle ticket to enter to win $50 Amazon Gift Card
*Format:* Paper *Estimated Time to Complete:* 15 minutes
**Objective:** To evaluate the participant's level of breast massage expertise

Participant Name: ____________________________ Email: ______________________________________

**LiquidGoldConcept, LLC Release Form**
**Permission for Use of Answers in LGC Breast Massage & Hand Expression Questionnaire 2015**

For valuable consideration received, I, ____________(PRINT NAME HERE)_________________ hereby irrevocably grant to LiquidGoldConcept, LLC., its subsidiaries, affiliates, nominees, licensees, their successors and assigns, and those acting with its authority (hereinafter collectively referred to as "LiquidGoldConcept"), with respect to the answers I wrote in the LGC Sim Model Questionnaire 2015 (the "Answers") written by me, the unrestricted absolute, perpetual, worldwide right to:

_______ (INITIAL) (a) Reproduce, copy, modify, create derivatives in whole or in part, or otherwise use the Answers or any part thereof in combination with or as a composite of other matter, including, but not limited to, text, data, images, photographs, illustrations, animation and graphics, video or audio segments of any nature, in any media or embodiment, now known or hereafter to become known, for any purpose whatsoever, including commercial, and

_______ (INITIAL) (b) Use and permit to be used my name, whether in original or modified form, in connection with the Answers, as LiquidGoldConcept may choose, and

_______ (INITIAL) (c) Display, perform, exhibit, distribute, transmit or broadcast the Answers by any means now known or hereafter to become known.

I hereby waive all rights and release and discharge LiquidGoldConcept from, and shall neither sue nor bring any proceeding against any such parties for, any claim, demand or cause of action whether now known or unknown, for defamation, invasion of right to privacy, publicity or personality or any similar matter, or based upon or relating to the use and exploitation of the Answers.

I certify that I am over the age of eighteen (18) years.

Dated: ____________________________________________

Name______________________________________________

Signature___________________________________________

**A:** *The following are questions regarding your experience working with* *<u>breastfeeding patients</u>. If you do not work with breastfeeding patients, you may skip this part and continue to Section B (Question 4).*

**1a**. Have you ever **expressed** a patient's breast milk with your hands?

- ☐ Yes
- ☐ No (If no, skip to 1c)
- ☐ Don't Know

**1b.** If yes, for what percentage of patients do you use your hands to **express** their breast milk?

- ☐ 25%
- ☐ 50%
- ☐ 75%
- ☐ 100%
- ☐ Other________________________

(Skip to 2a)

**1c.** If no, why not? Check all that apply.

- ☐ I don't know how.
- ☐ I don't think it is helpful.
- ☐ I think that it is gross
- ☐ My patients think it is gross
- ☐ Other________________________

**3e.** What teaching materials do you use for hand/manual **expression** of breast milk? Check all that apply.

- ☐ The patient's breast
- ☐ You own breasts
- ☐ A 2D picture
- ☐ A video
- ☐ A website
- ☐ An online forum
- ☐ A community group (Please, explain below)
- ☐ A single breast model (Please, explain below)
- ☐ A simulation model in the shape of a torso
- ☐ Other ________________

**3f.** What teaching materials do you use for hand/manual breast **massage?** Check all that apply.

- ☐ The patient's breast
- ☐ Your own breasts
- ☐ A 2D picture
- ☐ A video
- ☐ A website
- ☐ An online forum
- ☐ A community group (Please, explain below)
- ☐ A single breast model (Please, explain below)
- ☐ A simulation model in the shape of a torso
- ☐ Other ________________

**B.** *The following are questions regarding your experience* ***teaching health professional students and practitioners*** *about breastfeeding. If you do not teach students and practitioners, please, skip this part and continue to Part C (Question 7)*

**6a.** What tools do you use to teach about breastfeeding? Check all that apply.

- ☐ PowerPoint Presentation
- ☐ Printed Material (i.e. brochures, pamphlets)
- ☐ Multimedia (i.e. videos)
- ☐ Smartphone App
- ☐ Simulation Model (i.e. silicone breasts, mannequins)
- ☐ Standardized patient
- ☐ Real patient
- ☐ Other (Describe in 6b)

**6b.** Please use the space below to describe what tools you use?

**Part C.** *The following are questions regarding your **personal breastfeeding experience**. If you have not breastfed, please, skip this part and continue to Part D (Question 12).*

**7.** Please, describe the breastfeeding support you received in the hospital following delivery. Check all that apply.

- ☐ Lactation Consultant
- ☐ Support from the physician
- ☐ Support from the nurse (not a Lactation Consultant)
- ☐ Brochures/pamphlets
- ☐ A smartphone/tablet app
- ☐ Information about community support groups (ex. LaLecheLeague, WIC)
- ☐ Website or online support
- ☐ Breastfeeding Education Videos
- ☐ I didn't receive any breastfeeding support.
- ☐ I didn't deliver in a hospital

**8.** Please, describe the breastfeeding support you received from your family. Check all that apply.

- ☐ A member of my family positively impacted my decision to breastfeed.
- ☐ My parents were supportive of my choice to breastfeed my child.
- ☐ My in-laws were supportive of my choice to breastfeed my child.
- ☐ My husband/partner was supportive of my choice to breastfeed my child.

**9.** Did you use a breast pump?

- ☐ Yes
- ☐ No
- ☐ Don't Know

**10a**. Did you hand express your breast milk?

- ☐ Yes
- ☐ No
- ☐ Don't Know

**10b**. If yes to 10a, please explain how you learned to hand **express** your milk.

**11a.** Did you **massage** your breasts with your hands?

- ☐ Yes
- ☐ No
- ☐ Don't Know

11b. What breastfeeding concern prompted you to **massage your breasts** with your hands? Check all that apply.

- ☐ Breast pain
- ☐ Breast tenderness
- ☐ Engorgement
- ☐ Low milk supply
- ☐ Mastitis
- ☐ Nipple pain
- ☐ Oversupply of milk
- ☐ Plugged duct
- ☐ Poor milk flow
- ☐ Other (Please, describe)

**Part D.** *The following are demographic questions.*

12.Please describe your occupation and level of education (i.e. PhD, BA, DO etc.). If you are a healthcare professional, please list your specialty (i.e. pediatrics).

13 What is your age?

12. What is your race/ethnicity?

14. What is your gender?

15. Are you interested in participating in future surveys with LiquidGoldConcept, LLC?

- ☐ Yes
- ☐ No

**This is the end of the LGC Breast Massage & Hand Expression Questionnaire 2015.**

**Thank you very much for sharing your knowledge and your time.**

*Target Participant:* Breastfeeding Expert
*Reimbursement:* One raffle ticket to enter to win a $50 Amazon Gift Card
*Format:* Paper
*Estimated Time to Complete:* 20 minutes

**Objective:** To learn from the participant what features should be incorporated into a lactation simulation model

Participant Name: ___________________________ Email: ______________________________________

**LiquidGoldConcept, LLC Release Form**

**Permission for Use of Answers in LGC Sim Model Questionnaire 2016**

For valuable consideration received, I, ____________(PRINT NAME HERE)________________ hereby irrevocably grant to LiquidGoldConcept, LLC., its subsidiaries, affiliates, nominees, licensees, their successors and assigns, and those acting with its authority (hereinafter collectively referred to as "LiquidGoldConcept"), with respect to the answers I wrote in the LGC Sim Model Questionnaire 2015 (the "Answers") written by me, the unrestricted absolute, perpetual, worldwide right to:

_______ (INITIAL) (a) Reproduce, copy, modify, create derivatives in whole or in part, or otherwise use the Answers or any part thereof in combination with or as a composite of other matter, including, but not limited to, text, data, images, photographs, illustrations, animation and graphics, video or audio segments of any nature, in any media or embodiment, now known or hereafter to become known, for any purpose whatsoever, including commercial, and

_______ (INITIAL) (b) Use and permit to be used my name, whether in original or modified form, in connection with the Answers, as LiquidGoldConcept may choose, and

_______ (INITIAL) (c) Display, perform, exhibit, distribute, transmit or broadcast the Answers by any means now known or hereafter to become known.

I hereby waive all rights and release and discharge LiquidGoldConcept from, and shall neither sue nor bring any proceeding against any such parties for, any claim, demand or cause of action whether now known or unknown, for defamation, invasion of right to privacy, publicity or personality or any similar matter, or based upon or relating to the use and exploitation of the Answers.

I certify that I am over the age of eighteen (18) years.

Dated: ____________________________________________

Name______________________________________________

Signature___________________________________________

**Part A.** The following questions aim to understand what features you would like to see and feel in a lactation simulation model.

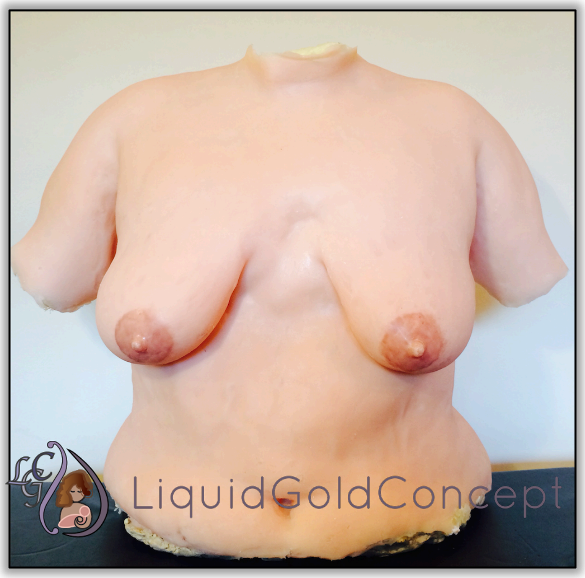


The above image is a proof-of-concept of the LiquidGoldConcept lactation simulation model we displayed at South by Southwest in Austin, TX in March 2015. In order to design the next prototype, <u>we need to know what features breastfeeding experts and educators want in a lactation simulation model.</u>

| | Very Valuable | | | | | | Not valuable |
|---|---|---|---|---|---|---|---|
| **1.** Please rate the value of **any** lactation simulation model as a training tool. | 7 | 6 | 5 | 4 | 3 | 2 | 1 |

Please explain your rating.

| | Very Relevant | | | | | | Not Relevant |
|---|---|---|---|---|---|---|---|
| **2.** Please rate the relevance of **any** lactation simulation model to your practice. | 7 | 6 | 5 | 4 | 3 | 2 | 1 |

| Please, explain your rating. |
|---|
| |

**3.** Which of the following is important to be able to do with a lactation simulation model? Check all that apply.

| # | Procedure | Yes | No |
|---|---|---|---|
| 1 | Ability to perform or teach the procedure of hand expression of breast milk | | |
| 2 | Ability to teach latch positions | | |
| 3 | Ability to attach a breast pump, turn it on, and demonstrate its function | | |
| 4 | Ability to teach simultaneous hand massage with breast pump | | |
| 5 | Ability to perform or teach reverse pressure softening | | |
| 6 | Ability to perform ultrasound to detect an abscess, or to treat plugged ducts | | |
| 7 | Ability to detect breast tenderness or engorgement | | |
| 8 | Ability to detect warmer areas of the breast | | |
| 9 | Ability to detect redness of "skin" | | |
| 10 | Ability to identify cracked, sore, bleeding nipples | | |
| 11 | Ability to identify milk blister or milk bleb | | |
| 12 | Ability to demonstrate skin to skin | | |
| 13 | Ability to detect Raynaud's Phenomenon | | |
| 14 | Ability to identify thrush | | |
| 15 | Ability to identify flat or inverted nipples | | |
| 16 | Ability to protract inverted nipples | | |
| 17 | Ability to detect insufficient glandular tissue | | |
| 19. | Ability to perform breast massage techniques | | |

| | |
|---|---|
| 18 | Other, Please describe any other techniques or procedures you would like to be able to do or teach with a lactation simulation model. |

**4.** Would you like to see/feel the following features in a lactation simulation model? Check all that apply.

| # | Features | Yes | No |
|---|---|---|---|
| 1 | Variety of breast shapes and sizes | | |
| 2 | Variety of nipple shapes and sizes | | |
| 3 | Breast reduction surgery | | |
| 4 | Breast augmentation surgery | | |
| 5 | Mastectomy | | |
| 6 | Areolar hair | | |
| 7 | Pierced nipples | | |
| 8 | Inverted Nipples | | |
| 9 | Milk or fluid comes out of nipple after stimulation | | |
| 10 | Difference in feel between breast, chest, and axillary tissue | | |
| 11 | Variety of skin colors | | |
| 12 | Variety of body shapes | | |
| 13 | Sim model with a head | | |
| 14 | Sim model with arms, legs, pelvis | | |
| 15 | Sim model as torso (see image above) | | |
| 16 | Sim model as just breasts without axilla | | |
| 17 | Electronic component (model responds and talks) | | |
| 18 | Virtual reality component (put on goggles) | | |
| 19 | Sim model as a wearable “apron” for standardized patient | | |

19. Other, please describe any other features you would like to see/feel in a lactation simulation model.

**5.** *The following are demographic questions.*

12.Please describe your occupation and level of education (i.e. PhD, BA, DO etc.). If you are a healthcare professional, please list your specialty (i.e. pediatrics).

13 What is your age?

12. What is your race/ethnicity?

14. What is your gender?

15. Are you interested in participating in future surveys with LiquidGoldConcept, LLC?

- ☐ Yes
- ☐ No

**This is the end of the LGC Sim Model Questionnaire 2016.**

**Thank you very much for sharing your knowledge and your time.**
