# Supplementary material for: Development and evaluation of a high-fidelity lactation simulation model for health professional breastfeeding education
Source: Int Breastfeed J. 2020 Feb 17;15:8. doi: 10.1186/s13006-020-0254-5 (PMC7026968; doi:10.1186/s13006-020-0254-5)
Supplement: Supplementary file 2 — Additional file 2. Physician resident survey (2017). Participant personal and professional background LSM validation questionnaire used with obstetrics and gynecology and family medicine residents. [file 13006_2020_254_MOESM2_ESM.pdf]

LiquidGoldConcept

ID: ____________

Thank you for participating in this series of questionnaires. Your feedback is very important to us. The information you provide will assist LiquidGoldConcept, a breastfeeding education technology startup, with the development of the Lactation Simulation Model (LSM) for use in lactation education.

This series of questionnaires will be distributed at the beginning, middle and end of the workshop. The questionnaires are optional and your answers are anonymous. Your choice to participate in any of the questionnaires will not affect your evaluation in this workshop nor will information from this questionnaire be used to measure your performance in this workshop. You do not have to complete any of the questionnaires or answer any question that makes you feel uncomfortable. Each questionnaire will take approximately 5-10 minutes of your time.

Validation Phase 1: Pre-Workshop Questionnaire

1. How do you self-identify? **Male** **Female** **Other __________**

2. Do you have personal experience with breastfeeding?
   - ☐ **No**
   - ☐ **Yes**, I have previously breastfed an infant.
   - ☐ **Yes**, I have assisted my partner with breastfeeding an infant.
   - ☐ **Other** (please describe) ___________________________________

3. Have you ever performed a prenatal breast exam? **Yes / No**

4. How often do you provide breastfeeding education to your pregnant patients?

   ☐ **None** of the time ☐ **Some** of the time ☐ **All** of the time

The next two questions (5, 6) involve 1-7 point scales. ***Choose a number between 1 and*** *7.*
Use the following scale to make a decision.

1	2	3	4	5	6	7
Strongly disagree	Moderately disagree	Mildly Disagree	Neither	Mildly Agree	Moderately Agree	Strongly Agree

5. "I am confident in my ability to perform a prenatal breast exam." ________

6. "I am confident in my ability to provide breastfeeding education to my patients."________

LiquidGoldConcept **ID: ____________**

Validation Phase 1: Intra-Workshop Questionnaire

Questions 1-3 listed below will address your opinion regarding the appearance of the Lactation Simulation Model (LSM). Before using or touching the model, visually inspect the LSM.

1. Do you agree or disagree with the following statements about the appearance of the LSM compared to a real patient. Use the following scale to make a decision.

1	2	3	4	5	6	7
Strongly disagree	Moderately disagree	Mildly Disagree	Neither/Not sure	Mildly Agree	Moderately Agree	Strongly Agree

	**1**	**2**	**3**	**4**	**5**	**6**	**7**
**A.** The model looks like a pregnant mother's chest.							
**B.** The breast size looks realistic.							
**C.** The breast shape looks realistic.							
**D**. The skin color looks realistic.							
**E.** The skin texture looks realistic.							
**F.** The **right** areola color looks realistic.							
**G**. The **left** areola color looks realistic.							
**H.** The **right** areola shape looks realistic.							
**I.** The **left** areola shape looks realistic.							
**J.** The size of the areolas (both) is realistic.							
**K.** The nipples look realistic (size and shape).							
**L**. The **right** nipple color looks realistic.							
**M**. The **left** nipple color looks realistic.							

At this point, the facilitators will lift the breasts for you to inspect underneath the breasts. The ***next 2 questions*** ***(2 and 3a)*** *ask about the LSM's ability to visually demonstrate different medical pathologies of the breast.*

2. Use descriptive language (eg. colors, patterns, textures, location, clinical presentation) to describe what you see on the left nipple. Write up to 30 words. *Example: At 3 o'clock, midway down, there is a bluish-purple discoloration that reminds me of a bruise.*

3a. Which of the following skin findings on the breasts, areolas, or nipples did you observe?

i. Do you observe any benign moles? **Yes / No**

a. If YES, how many?__________

b. If YES, where?____

____

ii. Do you observe any abnormal or potentially malignant moles? **Yes / No**

a. If YES, how many?__________

b. If YES, where?____

____

iii. Do you observe any freckles? **Yes / No**

iv. Do you observe any scars? **Yes / No**

a. If YES, how many? _________

b. If YES, where?____

____

c. If YES, describe the scar(s)? *Example: A 2-inch long erythematous scar in left axilla, in an early stage of wound healing by primary intention.*

____

____

____

v. Do you observe a nipple fissure? **Yes / No**

a. If YES, which nipple? **Right / Left**

b. If YES, where?____

____

vi. Do you observe any stretch marks? **Yes / No**

a. If YES, where?____

____

vii. Do you observe an(y) accessory nipple(s)? **Yes / No**

a. If YES, how many and where?

____

____

ID: ____________

3b. Please rate the following statement using the scale below as a guide:

1	2	3	4	5	6	7
Strongly disagree	Moderately disagree	Mildly Disagree	Neither/Not sure	Mildly Agree	Moderately Agree	Strongly Agree

"I am confident in my responses to questions 3a-i through 3a-vii." _________

Questions 4 and 5 will evaluate how realistic the LSM feels to the user. Prior to answering the questions, perform a breast exam on the LSM.

4. Describe whether you agree or disagree with the following statements about the feel of the LSM compared a real patient. Use the following scale to make a decision.

1	2	3	4	5	6	7
Strongly disagree	Moderately disagree	Mildly Disagree	Neither/Not sure	Mildly Agree	Moderately Agree	Strongly Agree

	**1**	**2**	**3**	**4**	**5**	**6**	**7**
**A.** The **right** breast feels realistic.							
**B.** The **left** breast feels realistic.							
**C.** The **right** nipple felt realistic.							
**D.** The **left** nipple felt realistic.							

ID: ____________

5. Without discussing with the group, perform a breast exam and identify <u>at least three</u> abnormal or unusual findings. There are <u>three superficial</u> (skin) and <u>three deep pathologies</u> present on the model. When you identify a pathology, sketch its location and describe *what it feels* like and/or *what it looks* like. *Example: A starburst pattern, hard nodule at 2 o'clock on right breast about 2 inches away from nipple.*

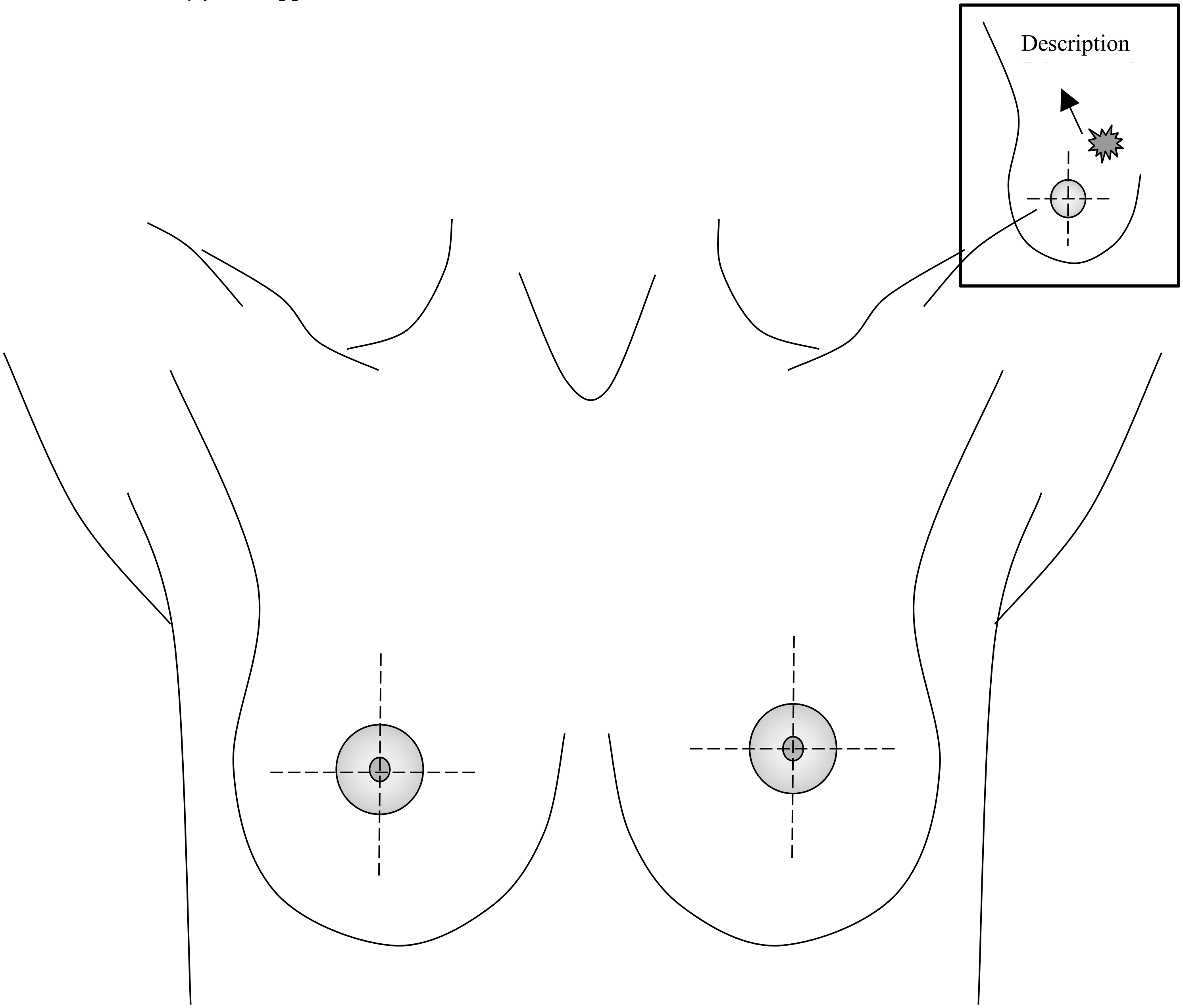

LiquidGoldConcept

ID: ____________

Validation Phase 1: Post-Test Questionnaire

The questions listed below address your overall experience with the LSM.

1. Describe whether you agree or disagree with the following statements about your ability to learn how to perform a breast exam using the LSM.
Use the following scale to make a decision.

1	2	3	4	5	6	7
Strongly disagree	Moderately disagree	Mildly Disagree	Neither/Not sure	Mildly Agree	Moderately Agree	Strongly Agree

	**1**	**2**	**3**	**4**	**5**	**6**	**7**
**A.**The model allowed me to practice comfortable positioning and movement *of my hands* during the breast exam.							
**B.** The model allowed me to practice comfortable positioning and movement *of my body* during the breast exam.							
**C.** The way I approached and interacted with the model was similar to how I (would) approach and interact with patients.							
**D.** The model helped me learn how to perform a prenatal breast exam.							

2. What features or improvements would you like to see incorporated into the model?

LiquidGoldConcept

ID: ___________

3.Please, tell us what you liked or did not like about the model.

Liked:

Did not like:

This is the end of the questionnaire series. Thank you for taking the time to provide feedback to LiquidGoldConcept about the LSM. The information you provide will assist LiquidGoldConcept in the development of this educational tool.

If you have questions or concerns regarding these questionnaires you may contact:

Anna Sadovnikova, MPH, MA
anna@liquidgoldconcept.com
