# Supplementary material for: Development and evaluation of a high-fidelity lactation simulation model for health professional breastfeeding education
Source: Int Breastfeed J. 2020 Feb 17;15:8. doi: 10.1186/s13006-020-0254-5 (PMC7026968; doi:10.1186/s13006-020-0254-5)
Supplement: Supplementary file 3 — Additional file 3. Nurse-midwifery student survey (2017). Participant personal and professional breastfeeding background and LSM validation questionnaire used with nurse-midwifery students. [file 13006_2020_254_MOESM3_ESM.pdf]

Date: ___________ ID___________

<u>Survey 1. Demographics and Breastfeeding Background</u>

1. How do you self-identify? ☐ Male ☐ Female ☐ Other________

2. How old are you?

☐ 18-24	☐ 36-40	☐ 51-55
☐ 25-30	☐ 41-45	☐ 56-60
☐ 31-35	☐ 46-50	☐ >60

3. Do you have clinical/community breastfeeding experience? ☐ Yes ☐ No ☐ Not sure

    a. *If you answered Yes to Question 3,* How many years of practice? _______
    b. *If you answered Yes to Question 3,* Please, describe your experience.

4. Do you have personal experience with breastfeeding?
    - ☐ ☐ No
    - ☐ ☐ Yes, I have previously breastfed an infant.
    - ☐ ☐ Yes, I have assisted my partner with breastfeeding an infant.
    - ☐ ☐ Other (please describe) ___________________________________

5. Have you performed a prenatal breast exam? ☐ Yes ☐ No ☐ Not sure

6. Have you performed a postpartum breast exam? ☐ Yes ☐ No ☐ Not sure

7. Have you provided breastfeeding education to a patient? ☐ Yes ☐ No ☐ Not sure

8. Have you ever worked with a breast simulation model? ☐ Yes ☐ No ☐ Not sure
    a. *If you answered Yes to Question 8,* please, describe the model to the best of your ability and the setting in which you used the model.

End of Survey 1

For questions or comments about the study sponsorship or design, contact Anna Sadovnikova:
anna@liquidgoldconcept.com (916) 905 3544

Date: ___________ ID___________

Survey 3. Evaluation of Look, Feel, and Realism of LSM

1. **Which LSM are you evaluating? Circle one.** Light / Dark

2. **Do you agree with the following statements? Use scale provided below.**

1	2	3	4	5	6	7
Strongly disagree	Disagree	Somewhat Disagree	Neither/Not sure	Somewhat Agree	Agree	Strongly Agree

Statements	1	2	3	4	5	6	7
1. The LSM looks like a breastfeeding mother's chest.							
2. The breast size looks realistic.							
3. The breast shape looks realistic.							
4. The skin color looks realistic.							
5. The skin texture looks realistic.							
6. The right areola color looks realistic.							
7. The left areola color looks realistic.							
8. The right areola shape looks realistic.							
9. The left areola shape looks realistic.							
10. The size of the areolas (both) is realistic.							
11. The nipple size looks realistic.							
12. The nipple shape looks realistic.							
13. The right nipple color looks realistic.							
14. The left nipple color looks realistic.							
15. The way the breast tissue moves into the breast pump looks realistic.							
16. The way simulated fluid comes out of the LSM is realistic.							
17. The engorgement looks realistic.							
18. The LSM allowed me to practice comfortable positioning and movement of my hands during the breast exam.							
19. The LSM allowed me to practice comfortable positioning and movement of my body during the breast exam.							
20. The way I approached and interacted with the LSM was similar to how I (would) approach and interact with patients.							
21. The LSM helped me learn how to perform a prenatal breast exam.							

For questions or comments about the study sponsorship or design, contact Anna Sadovnikova:
anna@liquidgoldconcept.com (916) 905 3544

Date: ___________ ID___________

3. What features or improvements would you like to see incorporated into the LSM?

4. What did you like about the LSM?

5. What did you dislike about the LSM?

End of Survey 3

For questions or comments about the study sponsorship or design, contact Anna Sadovnikova:
anna@liquidgoldconcept.com (916) 905 3544

Date: ___________ ID___________

Worksheet 1. Breastfeeding Basics

Student Instructions: Fill out this worksheet during Cases 1 through 4.

Case 1. "My breasts hurt"

For this case, only practice with one LSM. Make sure both breasts are engorged.

1. **Which LSM are you using?** Light / Dark / Both

2. **Demonstrate Reverse Pressure Softening (RPS) Technique.**

3. **Demonstrate at least one massage technique, other than RPS, to alleviate engorgement.**

Make sure you have a stopwatch/timer and a spoon. Perform hand expression for 15 seconds into a teaspoon. Count the number of drops.

4. **Which breast are you using?** R / L

5. **Were you able to hand express 5 drops or more in 15 seconds?** Yes / No / Not sure

6. **Have you ever received formal instruction on hand expression?** Yes / No / Not sure

7. **Have you ever expressed a patient's breast milk by hand?** Yes / No / Not sure

End of Case 1

Date: ___________ ID___________

Case 2. Breastfeeding hurts

For this case, please, examine both LSMs.

1. **Which of the following did you observe? Check all that apply. Circle Right (R) or Left (L), depending on where you observe the finding.**

Light LSM

Lipstick shaped nipple R / L

Round nipple R / L

Bulbous nipple R / L

Elongated nipple R / L

Inverted nipple R / L

Flat nipple R / L

Compression fissure, tip of nipple R / L

Compression fissure, base of nipple R / L

Bleeding nipple R / L

Cracked nipple R / L

Sore nipple R / L

Vasospasm R / L

Milk bleb R / L

Yeast infection on nipple R / L

Dark LSM

Lipstick shaped nipple R / L

Round nipple R / L

Bulbous nipple R / L

Elongated nipple R / L

Inverted nipple R / L

Flat nipple R / L

Compression fissure, tip of nipple R / L

Compression fissure, base of nipple R / L

Bleeding nipple R / L

Cracked nipple R / L

Sore nipple R / L

Vasospasm R / L

Milk bleb R / L

Yeast infection on nipple R / L

2. Demonstrate on the LSM where the newborn's mouth and tongue should be positioned to ensure optimal transfer of breast milk and to reduce nipple pain.

End of Case 2

For questions or comments about the study sponsorship or design, contact Anna Sadovnikova:
anna@liquidgoldconcept.com (916) 905 3544

Date: ___________ ID___________

Case 3. Not enough milk

For this case, you can practice with one or both LSMs. Work as a group to answer question 3.

1. **Assemble a breast pump. Time yourself.**
   Were you able to assemble a breast pump in under 2 minutes? Yes / No / Not sure

2. **Attach the breast pump to the LSM. Make sure the nipple is centered. Discuss with your classmates whether a different flange size is appropriate.**

3. **Which pump and flange size are best-suited for each breast?**

LSM	Breast	Pump Type	Flange Size
Dark / Light	R / L		
Dark / Light	R / L		
Dark / Light	R / L		
Dark / Light	R / L		

Grab a stopwatch. Remove the breast pump and hand express from a different breast or LSM for 15 seconds. Count the number of drops.

4. **Which LSM are you using?** Light / Dark

5. **Which breast are you expressing?** R / L

6. **Were you able to express five drops or more in 15 seconds?** Yes / No / Not Sure

End of Case 3

For questions or comments about the study sponsorship or design, contact Anna Sadovnikova:
anna@liquidgoldconcept.com (916) 905 3544

Date: ___________ ID___________

Case 4. Oversupply

For this case, only practice with one LSM.

Perform a breast exam.

1. **Which LSM are you using?** Light / Dark

Visual Inspection:

2. **Did you observe both breasts simultaneously?** Yes / No / Not sure
3. **Did you look in the axillae?** Yes / No / Not sure
4. **Did you look under the breasts?** Yes / No / Not sure

Palpation:

1. **Did you palpate the nipples?** Yes / No / Not sure
2. **Did you palpate the areolae?** Yes / No / Not sure
3. **Did you palpate underneath both breasts?** Yes / No / Not sure
4. **Did you palpate the axillae?** Yes / No / Not sure

Case 4 continues on next page→

For questions or comments about the study sponsorship or design, contact Anna Sadovnikova:
anna@liquidgoldconcept.com (916) 905 3544

Date: ___________ ID___________

5. **What abnormalities did you notice on this LSM?** Use the figure below to document your findings. Write down your top differential diagnosis for each abnormal finding. Findings can be dermatological, surgical, deep tissue, anatomical, etc.

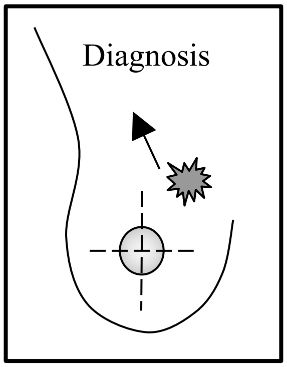

Example

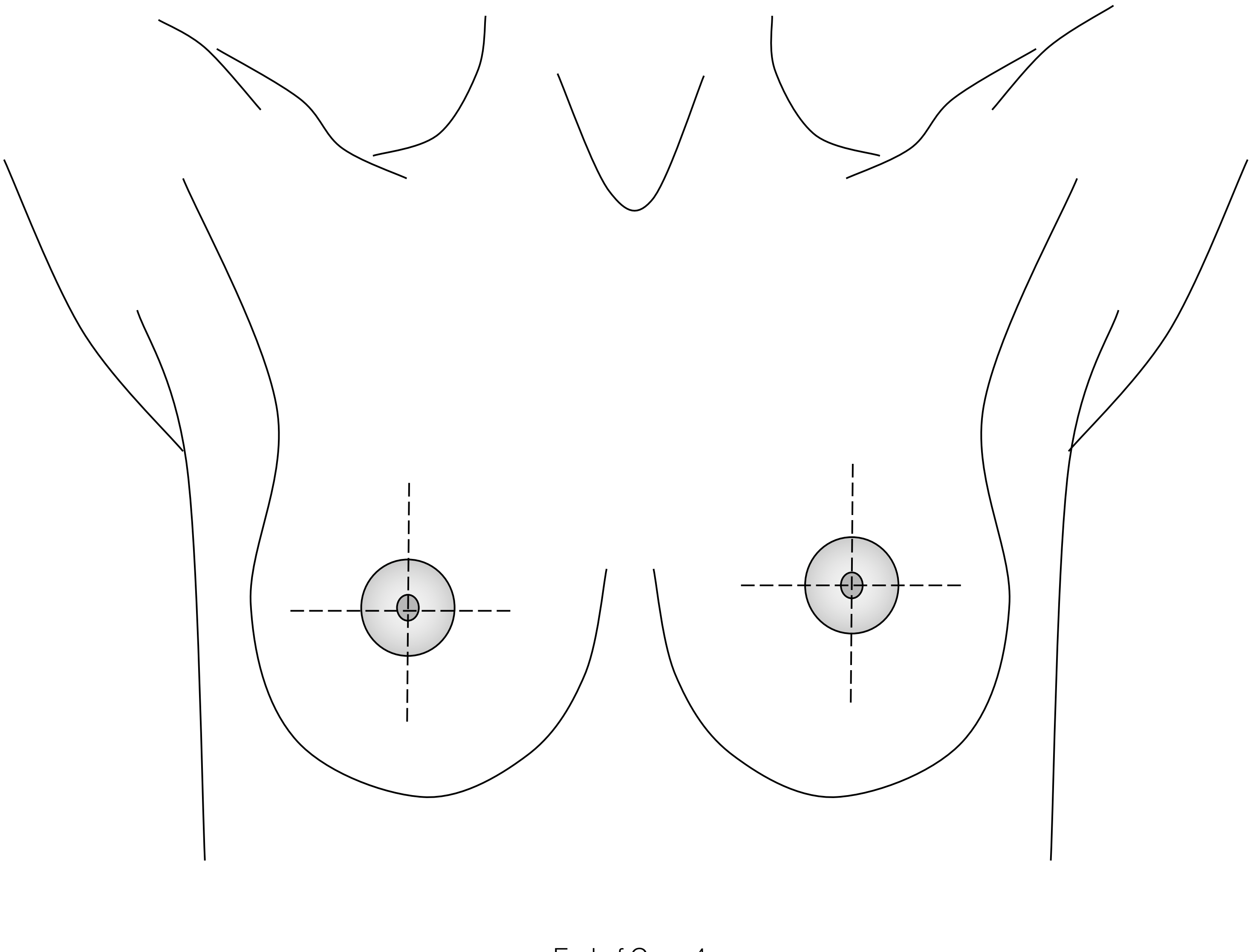

End of Case 4

For questions or comments about the study sponsorship or design, contact Anna Sadovnikova:
anna@liquidgoldconcept.com (916) 905 3544

Date: ___________ ID___________

Case 5. Breast Surgeries

Only use the Dark LSM for this case.

1. For each statement below, choose either True, False, or Not Sure.

Statements	True	False	Not Sure
1. The **left** breast has a scar in the inframammary fold.			
2. The **right** breast has a scar in the inframammary fold.			
3. The **left** breast has a periareolar scar.			
4. The **right** breast has a periareolar scar.			
5. The **left** breast has a breast biopsy scar.			
6. The **right** breast has a breast biopsy scar.			
7. I have seen (on real patients) breast reduction scars.			
8. I have seen (on real patients) breast augmentation scars.			
9. I have seen (on real patients) breast biopsy scars.			

2. Do you agree with the following statements? Use scale provided below.

1	2	3	4	5	6	7
Strongly disagree	Disagree	Somewhat Disagree	Neither/Not sure	Somewhat Agree	Agree	Strongly Agree

Statements	1	2	3	4	5	6	7
22. The inframammary scar looks realistic.							
23. The periareolar scar looks realistic.							
24. The biopsy scar looks realistic.							

3. If you gave any the scars a score of 6 or below, please, explain your rationale.

End of Case 5

For questions or comments about the study sponsorship or design, contact Anna Sadovnikova:
anna@liquidgoldconcept.com (916) 905 3544

Date: ___________ ID___________

Case 6. Ectopic Breast Tissue

Only use the Dark LSM for this case.

1. Do you agree with the following statements? Use scale provided below.

1	2	3	4	5	6	7
Strongly disagree	Disagree	Somewhat Disagree	Neither/Not sure	Somewhat Agree	Agree	Strongly Agree

Statements	1	2	3	4	5	6	7
1. The ectopic breast feels like breast tissue.							
2. The ectopic breast looks realistic.							
3. I can hand express simulated milk from the ectopic breast tissue in the DARK LSM.							
4. I am confident in my ability to counsel a patient on the management of ectopic breast tissue.							

2. For each statement below, choose either True, False, or Not Sure.

Statements	True	False	Not Sure
1. Ectopic breast tissue can only be found in the axilla.			
2. Polythelia is synonymous with supernumerary nipples.			
3. An ectopic breast has its own nipple.			
4. Because ectopic breast tissue is not connected to the ductal system, patients cannot develop mastitis in ectopic breast tissue.			

End of Case 6

For questions or comments about the study sponsorship or design, contact Anna Sadovnikova:
anna@liquidgoldconcept.com (916) 905 3544

Date: ___________ ID___________

Case 7. Breast Mass

Only use one LSM for this case. You do not need to switch after you are done.

1. Which LSM did you use? Light / Dark

2. Perform a breast exam. Draw in where you felt masses. Try to draw them true to size. If you feel a mass the size of a marble—draw a circle the size of a marble and describe how deep into the tissue you had to press to feel it (1/4 inch? 1 inch?) Describe the mass—is it round? Bumpy? Does it freely move within the tissue?

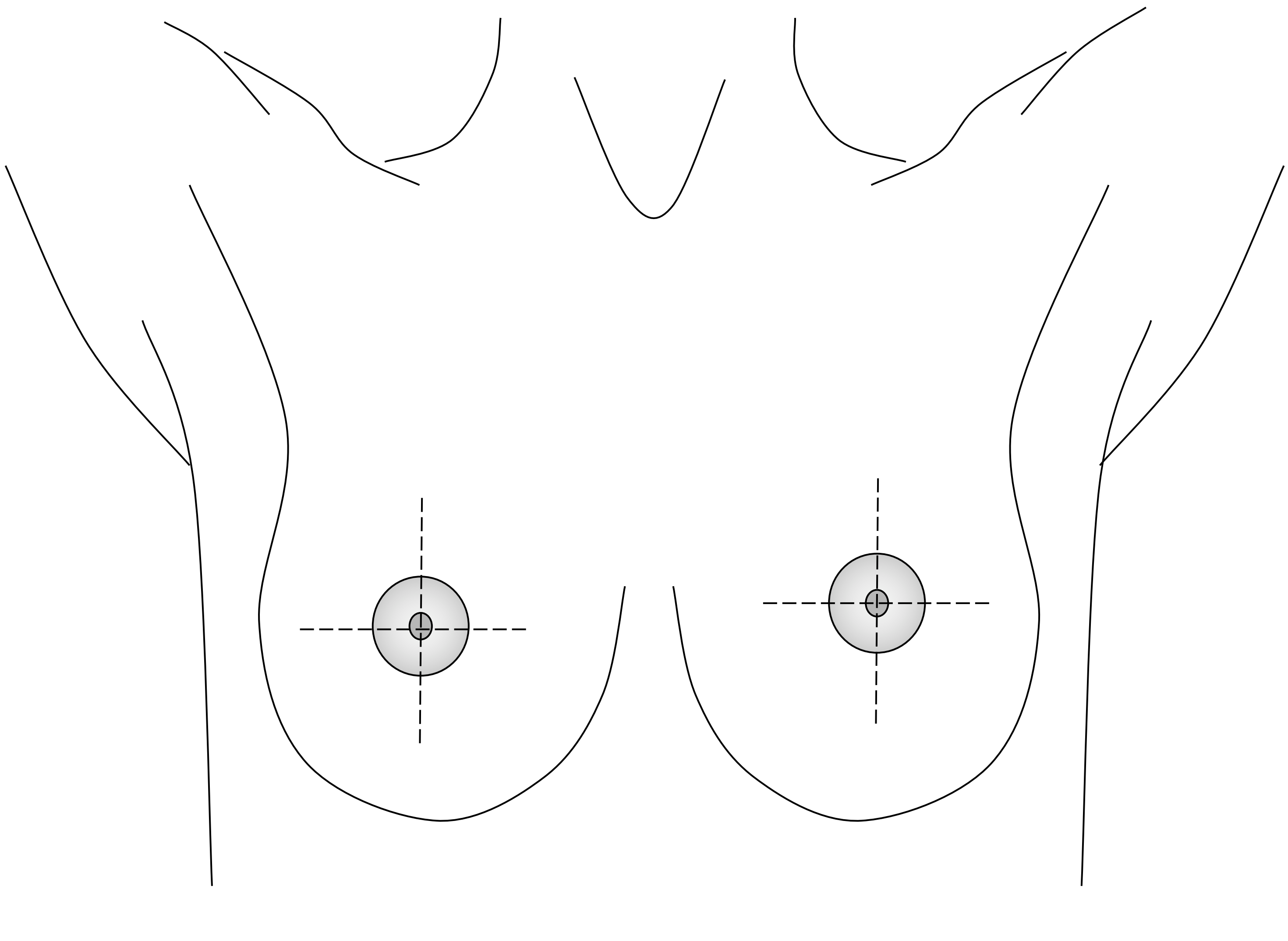

End of Case 7

For questions or comments about the study sponsorship or design, contact Anna Sadovnikova:
anna@liquidgoldconcept.com (916) 905 3544

Date: ____________ ID____________

Case 8. Mastitis

Make sure get a chance to examine both LSMs.

1. Use descriptive language (~20 words, size, color, pattern, shape, feel, etc.) to describe what you see on the left breast of the LIGHT LSM.

2. Use descriptive language (~20 words, size, color, pattern, shape, feel, etc.) to describe what you see on the left breast of the DARK LSM.

3. Do you agree with the following statements? Use scale provided below.

1	2	3	4	5	6	7
Strongly disagree	Disagree	Somewhat Disagree	Neither/Not sure	Somewhat Agree	Agree	Strongly Agree

Statements	1	2	3	4	5	6	7
1. The LIGHT LSM has early stage mastitis on the left breast.							
2. The DARK LSM has early stage mastitis on the left breast.							
3. The LIGHT LSM has late stage mastitis/abscess on the left breast.							
4. The DARK LSM has late stage mastitis/abscess on the left breast.							
5. The early stage mastitis looks realistic.							
6. The late stage mastitis looks realistic.							
7. I am confident in my responses to the previous 6 questions.							

Date: ___________ ID___________

End of Case 8

For questions or comments about the study sponsorship or design, contact Anna Sadovnikova:
anna@liquidgoldconcept.com (916) 905 3544
