# Supplementary material for: Development and evaluation of a high-fidelity lactation simulation model for health professional breastfeeding education
Source: Int Breastfeed J. 2020 Feb 17;15:8. doi: 10.1186/s13006-020-0254-5 (PMC7026968; doi:10.1186/s13006-020-0254-5)
Supplement: Supplementary file 4 — Additional file 4. Clinical lactation expert survey (2018). Participant personal and professional background and LSM validation questionnaire used with clinical lactation experts. [file 13006_2020_254_MOESM4_ESM.pdf]

Date: July 6-8, 2018 ID____________

Instructions

Thank you for agreeing to provide feedback and evaluate the LiquidGoldConcept Lactation Simulation Models.

The study coordinator has assigned a study identification number to you.

You have been randomly assigned to evaluate **either** <u>LSMs #1 and #2</u> **or** <u>LSMs #3 and #4.</u>

It will take you between 30 and 40 minutes to complete this survey packet.

The study coordinator cannot answer questions about the look, feel, or realism of the LSM. She/he can only provide information about the study logistics and/or clarify instructions.

Would like to be included in the LiquidGoldConcept, Inc. mailing list to receive information about curricular materials, research studies, and discounts?

- O **Yes**
- O **No**

Section 1. Demographics and Breastfeeding Background

1. **How do you self-identify?** O Male O Female O Other________
2. **What is your age? ______**
3. **What is your profession (choose all that apply)?**
   - O MD/DO/MBBS or equivalent
   - O Physician assistant
   - O Advanced practice nurse or equivalent
   - O IBCLC
   - O Other:_______________ ____________________
4. **What is your specialty?**
   - O Pediatrics
   - O Obstetrics and gynecology
   - O Family medicine
   - O Internal medicine
   - O Emergency medicine
   - O Surgery
   - O Midwifery
   - O Other___________
5. **Are you still in residency training?**
   - O Yes
   - O No
6. **Where do you practice?**
   - O United States (State:__________)
   - O Canada (Province:___________)
7. **Do you practice at an academic/teaching center?**
   - O Yes
   - O No
8. **How many years of practice in breastfeeding medicine? _________**

9. Did you obtain additional certification or training to work in breastfeeding medicine?

- O No
- O Yes, please mark all that apply:
  - o FABM
  - o IBCLC
  - o Certified lactation counselor, educator, or equivalent
  - o I am currently working toward IBCLC/CLC/equivalent
  - o Other:____

10. Do you have personal experience with breastfeeding?

- O No
- O Yes, I have previously breastfed.
- O Yes, I have assisted my partner with breastfeeding.
- O Other: ____

11. What percentage of your practice is with breastfeeding mothers?

- O 0-24%
- O 25-49%
- O 50-74%
- O 75-100%
- O Don't know.

12. Approximately what percentage of your breastfeeding patient population is white, non-Hispanic?

- O 0-24%
- O 25-49%
- O 50-74%
- O 75-100%
- O Don't know.

13. Have you worked with a breast model/simulator before?

- O No
- O If yes, circle the breast model(s)/simulator(s) you have worked with before. *If the breast model/simulator is not pictured below, please, describe the product you worked with to the best of your ability in the box on the right.*

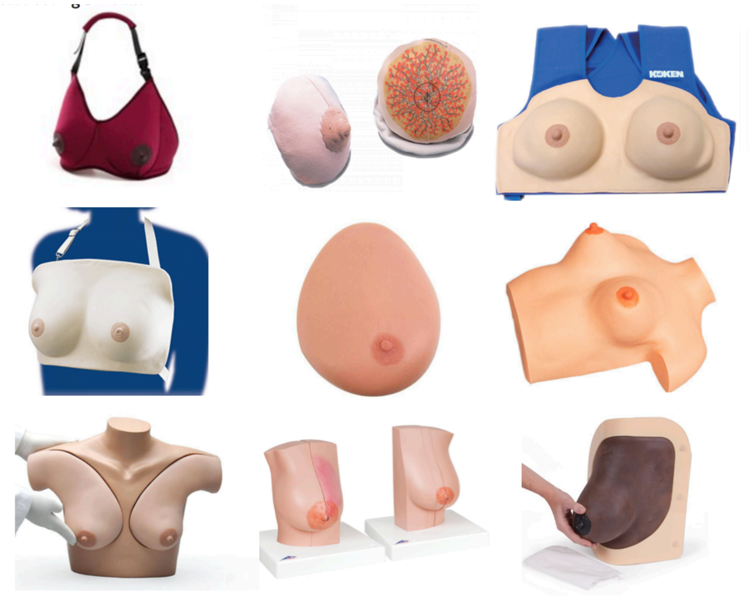

14. Have you seen the LiquidGoldConcept Lactation Simulation Model before?

- O No
- O If yes, please, explain:____

STOP. Turn in this Questionnaire Packet (Section 1) to the study coordinator.

Date: July 6-8, 2018 ID____________

Section 2a. Breast Exam and Documentation of Findings

You are now evaluating LSM # ____. Please, confirm that this is the LSM you are working with.

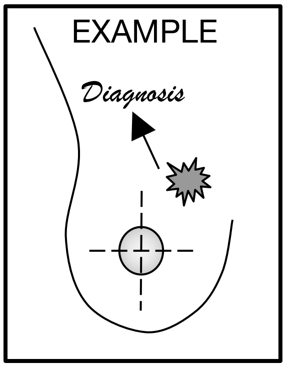

Instructions. Perform a breast exam. Use the figure below to document your findings. **Write down your top differential diagnosis** for each finding. Findings can be dermatological, surgical, deep tissue, anatomical, etc. *Make every effort to draw true to scale.*

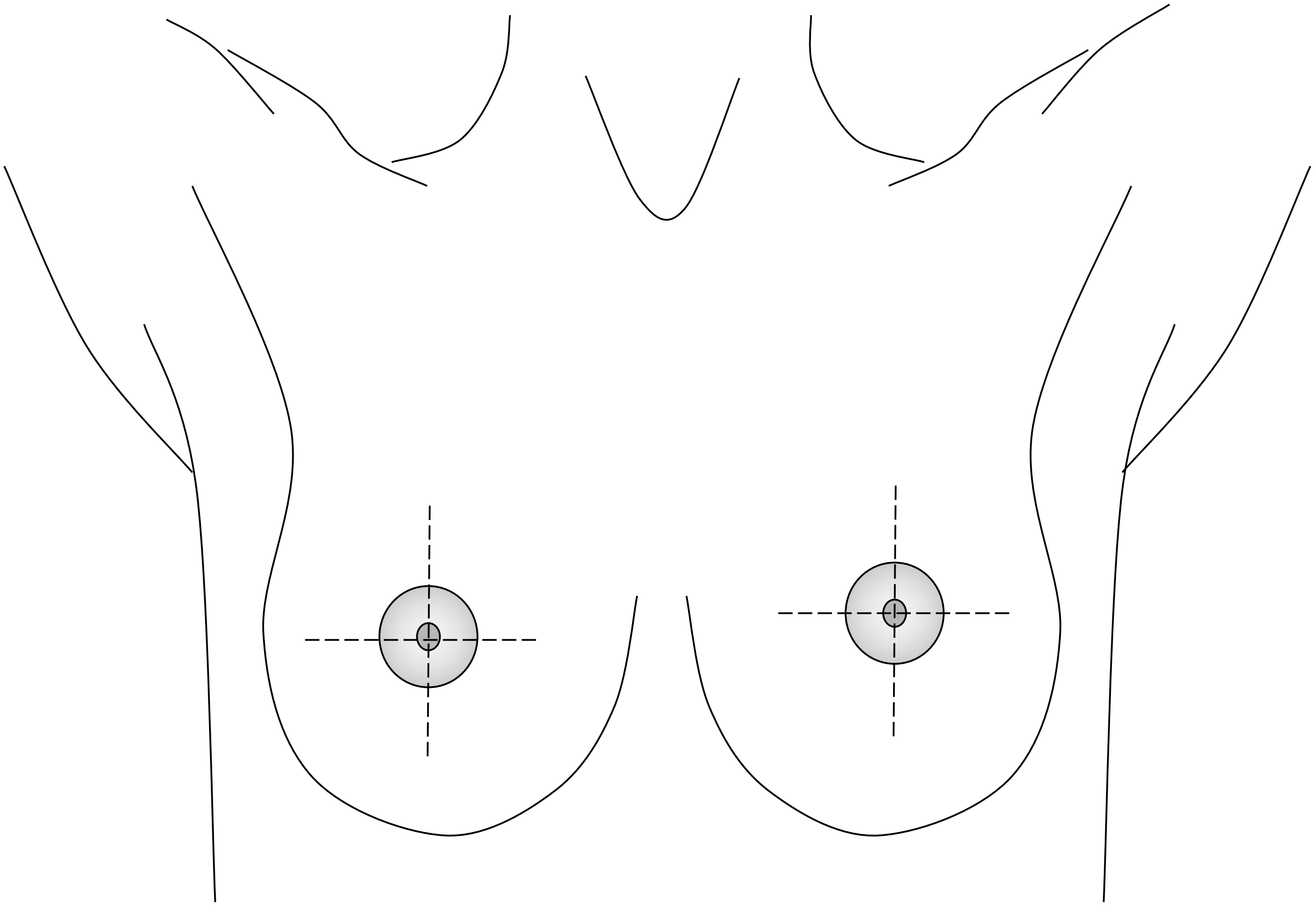

STOP. Turn this page in to the study coordinator. Then, proceed to the next section (Section 2a) of the survey.

Section 2b. Validation of the LSM Look, Feel, Pathologies, and Realism

You are evaluating LSM # ____. Please, confirm that this is the LSM you are working with.

Do you agree with the following statements? Use scale provided below.

1	2	3	4	5	6	7
Strongly disagree	Disagree	Somewhat Disagree	Neither/Not sure	Somewhat Agree	Agree	Strongly Agree

**Statements**	**1**	**2**	**3**	**4**	**5**	**6**	**7**
**General Look and Feel of LSM**							
1. The LSM looks like a breastfeeding mother's chest.							
2. The breast size looks realistic.							
3. The breast shape looks realistic.							
4. The skin color looks realistic.							
5. The skin feels realistic.							
***Right* Breast Look and Feel**							
1. The right areolar color looks realistic.							
2. The right areolar shape looks realistic.							
3. The right areolar size looks realistic.							
4. The right nipple color looks realistic.							
5. The right nipple feels realistic.							
6. The right nipple shape is realistic.							
7. The right nipple size is realistic.							
8. The right breast feels realistic.							
***Left* Breast Look and Feel**							
1. The left areolar color looks realistic.							
2. The left areolar shape looks realistic.							
3. The left areolar size looks realistic.							
4. The left nipple color looks realistic.							
5. The left nipple feels realistic.							
6. The left nipple shape is realistic.							
7. The left nipple size is realistic.							
8. The left breast feels realistic.							

Date: July 6-8, 2018 ID____________

1	2	3	4	5	6	7
Strongly disagree	Disagree	Somewhat Disagree	Neither/Not sure	Somewhat Agree	Agree	Strongly Agree

**Statements about Realism of Pathologies**	**1**	**2**	**3**	**4**	**5**	**6**	**7**
1. The plugged duct(s) in the left breast feel(s) realistic.							
2. The plugged duct(s) in the right breast feel(s) realistic.							
3. The mastitis looks realistic.							
4. The Montgomery glands look realistic.							
5. The damage on the left nipple looks realistic.							

**Realism of Breastfeeding Techniques and Experience**	**1**	**2**	**3**	**4**	**5**	**6**	**7**
**Instructions: Put on the LSM.<br>The study coordinator can help you with adjustment of straps.**							
Putting on the LSM is intuitive.							
Wearing the LSM is comfortable.							
The weight of the LSM is appropriate.							
**Instructions: Set the timer for 15 seconds.<br>Hand express colostrum from the *right* breast into the spoon.**							
The amount of simulated colostrum I hand expressed in 15 seconds is realistic.							
The way that simulated colostrum comes out of the nipple is realistic.							
**Instructions: Set the timer for 15 seconds.<br>Hand express colostrum from the *left* breast into the spoon.**							
The amount of simulated colostrum I hand expressed in 15 seconds is realistic							
The way that simulated colostrum comes out of the nipple is realistic.							

Date: July 6-8, 2018 ID____________

1	2	3	4	5	6	7
Strongly disagree	Disagree	Somewhat Disagree	Neither/Not sure	Somewhat Agree	Agree	Strongly Agree

**Instructions: Use the breast pump to express colostrum from the *right* breast.**							
What flange size did you use? *Write number to the right* →							
At what setting did you set the pump to observe a realistic movement of breast tissue? Please, describe.							
**Statements**	**1**	**2**	**3**	**4**	**5**	**6**	**7**
The way the breast tissue moves into the breast pump is realistic.							
The way simulated colostrum comes out of the breast is realistic.							

**Instructions: Use the breast pump to express colostrum from the *left* breast.**							
What flange size did you use? *Write number to the right* →							
At what setting did you set the pump to observe a realistic movement of breast tissue? Please, describe.							
**Statements**	**1**	**2**	**3**	**4**	**5**	**6**	**7**
The way the breast tissue moves into the breast pump is realistic.							
The way simulated colostrum comes out of the breast is realistic.							
**Instructions: Engorge *both* breasts using the black bulbs hanging from the LSM by twisting the knob clockwise. Hand express colostrum from *both* breasts. No need to set a timer.**							
**Statements**	**1**	**2**	**3**	**4**	**5**	**6**	**7**
The engorgement looks realistic.							
The engorgement feels realistic.							
The way that simulated colostrum comes out of the ***engorged right*** breast when I hand express is realistic.							
The way that simulated colostrum comes out of the ***engorged left*** breast when I hand express is realistic.							

Date: July 6-8, 2018 ID___________

1	2	3	4	5	6	7
Strongly disagree	Disagree	Somewhat Disagree	Neither/Not sure	Somewhat Agree	Agree	Strongly Agree

**Instructions: Remove the LSM and complete the rest of the questionnaire.**							
**Realism of Experience (continued)**	**1**	**2**	**3**	**4**	**5**	**6**	**7**
Removing the LSM from my body is intuitive.							
The LSM allowed me to practice correct positioning and movement of my hands.							
**This** LSM is a useful tool for health professional student education.							
**This** LSM is a useful tool for hospital staff education.							
**This** LSM is a useful tool for patient education.							

What features/improvements should be incorporated into LSM (#_____)?

What did you like about this LSM (#_____)?

What did you dislike about this LSM (#_____)?

STOP. Turn in this questionnaire packet (Section 2b) to the study coordinator before proceeding to the next LSM.

Section 3a. Breast Exam and Documentation of Findings

You are now evaluating LSM # ____. Please, confirm that this is the LSM you are working with.

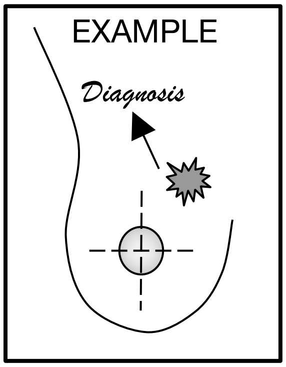

Instructions. Perform a breast exam. Use the figure below to document your findings. **Write down your top differential diagnosis** for each finding. Findings can be dermatological, surgical, deep tissue, anatomical, etc. *Make every effort to draw true to scale.*

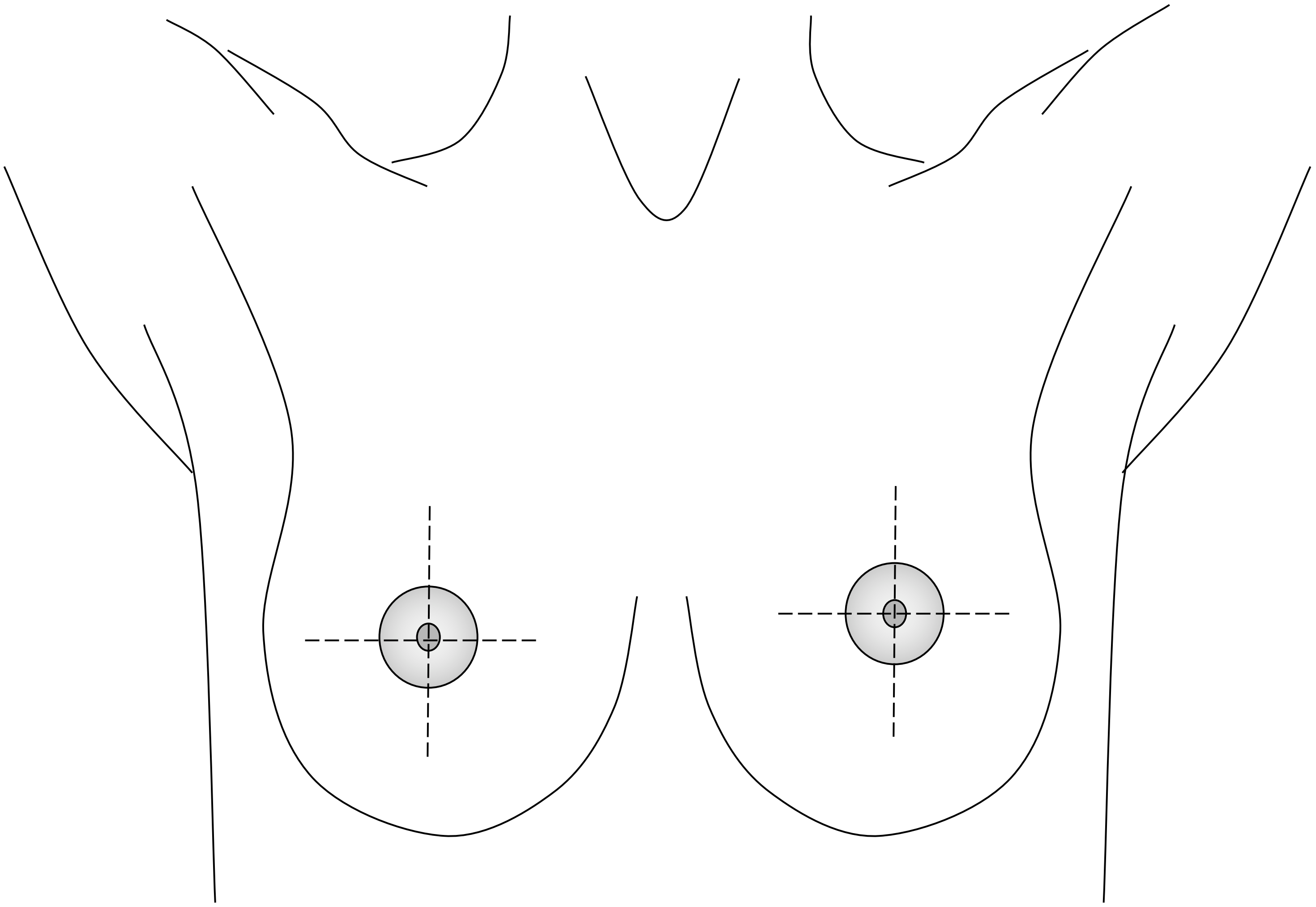

STOP. Turn this page in to the study coordinator. Then, proceed to the next section (Section 3a) of the survey.

Section 3b. Validation of the LSM Look, Feel, Pathologies, and Realism

You are evaluating LSM # ____. Please, confirm that this is the LSM you are working with.

1. Do you agree with the following statements? Use scale provided below.

1	2	3	4	5	6	7
Strongly disagree	Disagree	Somewhat Disagree	Neither/Not sure	Somewhat Agree	Agree	Strongly Agree

Statements	1	2	3	4	5	6	7
**General Look and Feel of LSM**							
6. The LSM looks like a breastfeeding mother's chest.							
7. The breast size looks realistic.							
8. The breast shape looks realistic.							
9. The skin color looks realistic.							
10. The skin feels realistic.							
***Right* Breast Look and Feel**							
9. The right areolar color looks realistic.							
10. The right areolar shape looks realistic.							
11. The right areolar size looks realistic.							
12. The right nipple color looks realistic.							
13. The right nipple feels realistic.							
14. The right nipple shape is realistic.							
15. The right nipple size is realistic.							
16. The right breast feels realistic.							
***Left* Breast Look and Feel**							
9. The left areolar color looks realistic.							
10. The left areolar shape looks realistic.							
11. The left areolar size looks realistic.							
12. The left nipple color looks realistic.							
13. The left nipple feels realistic.							
14. The left nipple shape is realistic.							
15. The left nipple size is realistic.							
16. The left breast feels realistic.							

1	2	3	4	5	6	7
Strongly disagree	Disagree	Somewhat Disagree	Neither/Not sure	Somewhat Agree	Agree	Strongly Agree

**Statements about Realism of Pathologies**	**1**	**2**	**3**	**4**	**5**	**6**	**7**
1. The plugged duct(s) in the left breast feel(s) realistic.							
2. The plugged duct(s) in the right breast feel(s) realistic.							
3. The abscess looks realistic.							
4. The milk bleb looks realistic.							
5. The damage on the left nipple looks realistic.							
6. The scar on the right breast looks realistic.							
7. The scar on the left breast looks realistic.							
8. The ectopic tissue looks realistic.							
9. The ectopic tissue feels realistic.							

**Realism of Breastfeeding Techniques and Experience**	**1**	**2**	**3**	**4**	**5**	**6**	**7**
**Instructions: Put on the LSM. The study coordinator can help you with adjustment of straps.**							
Putting on the LSM is intuitive.							
Wearing the LSM is comfortable.							
The weight of the LSM is appropriate.							
**Instructions: Set the timer for 15 seconds. Hand express colostrum from the *right* breast.**							
The amount of simulated colostrum I hand expressed in 15 seconds is realistic.							
The way that simulated colostrum comes out of the nipple is realistic.							
**Instructions: Set the timer for 15 seconds. Hand express colostrum from the *left* breast.**							
The amount of simulated colostrum I hand expressed in 15 seconds is realistic							
The way that simulated colostrum comes out of the nipple is realistic.							

Date: July 6-8, 2018 ID____________

1	2	3	4	5	6	7
Strongly disagree	Disagree	Somewhat Disagree	Neither/Not sure	Somewhat Agree	Agree	Strongly Agree

**Instructions: Use the breast pump to express colostrum from the *right* breast.**							
What flange size did you use? *Write number to the right* →							
At what setting did you set the pump to observe a realistic movement of breast tissue? Please, describe.							
**Statements**	**1**	**2**	**3**	**4**	**5**	**6**	**7**
The way the breast tissue moves into the breast pump is realistic.							
The way simulated colostrum comes out of the breast is realistic.							

**Instructions: Use the breast pump to express colostrum from the *left* breast.**							
What flange size did you use? *Write number to the right* →							
At what setting did you set the pump to observe a realistic movement of breast tissue? Please, describe.							
**Statements**	**1**	**2**	**3**	**4**	**5**	**6**	**7**
The way the breast tissue moves into the breast pump is realistic.							
The way simulated colostrum comes out of the breast is realistic.							
**Instructions: Engorge *both* breasts using the black bulbs hanging from the LSM by twisting the knob clockwise. Hand express colostrum from *both* breasts. No need to set a timer.**							
**Statements**	**1**	**2**	**3**	**4**	**5**	**6**	**7**
The engorgement looks realistic.							
The engorgement feels realistic.							
The way that simulated colostrum comes out of the ***engorged right*** breast when I hand express is realistic.							
The way that simulated colostrum comes out of the ***engorged left*** breast when I hand express is realistic.							

1	2	3	4	5	6	7
Strongly disagree	Disagree	Somewhat Disagree	Neither/Not sure	Somewhat Agree	Agree	Strongly Agree

**Instructions: Remove the LSM and complete the rest of the questionnaire.**							
**Realism of Experience (continued)**	**1**	**2**	**3**	**4**	**5**	**6**	**7**
Removing the LSM from my body is intuitive.							
The LSM allowed me to practice correct positioning and movement of my hands.							
**This** LSM is a useful tool for health professional student education.							
**This** LSM is a useful tool for hospital staff education.							
**This** LSM is a useful tool for patient education.							

What features/improvements should be incorporated into LSM (#_____)?

What did you like about this LSM (#_____)?

What did you dislike about this LSM (#_____)?

This is the end of Questionnaire. Thank you for your time and feedback! Please, return this survey packet to the study coordinator and confirm your email to receive the $10.00 Amazon gift card.
